# De Novo *DNM1L* Pathogenic Variant Associated with Lethal Encephalocardiomyopathy—Case Report and Literature Review

**DOI:** 10.3390/ijms26020846

**Published:** 2025-01-20

**Authors:** Martina Magistrati, Luisa Zupin, Eleonora Lamantea, Enrico Baruffini, Daniele Ghezzi, Andrea Legati, Fulvio Celsi, Flora Maria Murru, Valeria Capaci, Maurizio Pinamonti, Rossana Bussani, Marco Carrozzi, Cristina Dallabona, Massimo Zeviani, Maria Teresa Bonati

**Affiliations:** 1 Department of Chemistry, Life Sciences and Environmental Sustainability, University of Parma, Parco Area delle Scienze, 11/A, 43124 Parma, Italy; martina.magistrati@unipr.it (M.M.); cristina.dallabona@unipr.it (C.D.); 2 Institute for Maternal and Child Health IRCCS Burlo Garofolo, Via dell’Istria, 65, 34137 Trieste, Italy; luisa.zupin@burlo.trieste.it (L.Z.); fulvio.celsi@burlo.trieste.it (F.C.); floramaria.murru@burlo.trieste.it (F.M.M.); valeria.capaci@burlo.trieste.it (V.C.); marco.carrozzi@burlo.trieste.it (M.C.); massimo.zeviani@burlo.trieste.it (M.Z.); 3 Unit of Medical Genetics and Neurogenetics, Fondazione IRCCS Istituto Neurologico Carlo Besta, Via Celoria, 11, 20133 Milan, Italy; eleonora.lamantea@istituto-besta.it (E.L.); daniele.ghezzi@istituto-besta.it (D.G.); andrea.legati@istituto-besta.it (A.L.); 4 Department of Pathophysiology and Transplantation, University of Milan, Via F. Sforza, 35, 20122 Milan, Italy; 5 Institute of Pathological Anatomy and Histology, Azienda Sanitaria Universitaria Giuliano Isontina (ASUGI), University of Trieste, Via Giacomo Puccini, 50, 34148 Trieste, Italy; maurizio.pinamonti@asugi.sanita.fvg.it (M.P.); rossana.bussani@asugi.sanita.fvg.it (R.B.); 6 Department of Medical, Surgical and Health Sciences, University of Trieste, Via Giacomo Puccini, 50, 34148 Trieste, Italy

**Keywords:** *DNM1L*, lethal encephalocardiomyopathy, refractory status epilepticus (RSE), burst suppression, global developmental regression, hypertrophic cardiomyopathy (HC), retrospective post-mortem diagnosis

## Abstract

Pathogenic variants in *DNM1L*, encoding dynamin-like protein-1 (DRP1), cause a lethal encephalopathy. DRP1 defective function results in altered mitochondrial networks, characterized by elongated/spaghetti-like, highly interconnected mitochondria. We validated in yeast the pathogenicity of a de novo *DNM1L* variant identified by whole exome sequencing performed more than 10 years after the patient’s death. Meanwhile, we reviewed the broadness and specificities of *DNM1L*-related phenotype. The patient, who exhibited developmental delay in her third year, developed a therapy-refractory myoclonic status epilepticus, followed by neurological deterioration with brain atrophy and refractory epilepsy. She died of heart failure due to hypertrophic cardiomyopathy. She was found to be heterozygous for the *DNM1L* variant (NM_ 012062.5):c.1201G>A, p.(Gly401Ser). We demonstrated its deleterious impact and dominant negative effect by assessing haploid and diploid mutant yeast strains, oxidative growth, oxygen consumption, frequency of *petite*, and architecture of the mitochondrial network. Structural modeling of p.(Gly401Ser) predicted the interference of the mutant protein in the self-oligomerization of the DRP1 active complex. *DNM1L*-related phenotypes include static or (early) lethal encephalopathy and neurodevelopmental disorders. In addition, there may be ophthalmological impairment, peripheral neuropathy, ataxia, dystonia, spasticity, myoclonus, and myopathy. The clinical presentations vary depending on mutations in different DRP1 domains. Few pathogenic variants, the p.(Gly401Ser) included, cause an encephalocardiomyopathy with refractory status epilepticus.

## 1. Introduction

Heterozygous, mostly de novo, pathogenic variants in *DNM1L*, encoding dynamin-like protein-1 (DRP1), cause a lethal encephalopathy due to defective mitochondrial and peroxisomal fission 1 (EMPF1, OMIM #614388). However, bi-allelic inheritance was reported in a few patients with healthy heterozygous parents [1,2,3], and individuals from three unrelated French families harboring a heterozygous *DNM1L* variant were described to be affected by non-syndromic optic neuropathy (OPA5, OMIM #610708), exhibiting autosomal dominant inheritance with full penetrance [4,5].

DRP1 is a major component of the mitochondrial fission system. It is an evolutionarily conserved GTPase that forms concentric ring-like structures via self-homotetramerization that surrounds the scission site, followed by GTPase-dependent constriction [6]. Fission and fusion (i.e., ‘mitochondrial dynamics’) are continuous processes important for maintaining organelle function through the removal of damaged components, genetic complementation, distribution of the organelles during mitosis, and several other cellular functions. Elongated, spaghetti-like organelles are typically present in the cells of patients with *DNM1L* pathogenic variants.

DRP1 contains a N-terminal GTPase head, a middle domain (MD), important for tetramerization of the protein [7], and a C-terminal GTPase effector domain (GED), as well as a non-conserved Variable Domain (VD) [6].

Here, in the context of a retrospective research study, we report on the clinical history of a baby girl affected by a lethal encephalocardiomiopathy due to a de novo *DNM1L* variant (NM_ 012062.5):c.1201G>A, p.(Gly401Ser). Since the patient died 12 years before carrying out the whole exome sequencing, so her tissues and primary cells were not available, the yeast model was exploited to characterize the identified variant. We confirmed the pathogenicity of the p.(Gly401Ser) in yeast, demonstrating its deleterious impact on mitochondrial function and its dominant negative effect. Moreover, an extensive literature review allowed us to understand the broad spectrum of *DNM1L*-related disorders: alongside early lethality, refractory status epilepticus, or several neurological signs in patients exhibiting a static encephalopathy, *DNM1L* pathovariants may also occur in neurodevelopmental disorders or in isolated paroxysmal hemiparesis. Meanwhile, we found that cardiomyopathy was a cause of death. We suggest including *DNM1L* among genes of mitochondrial hypertrophic cardiomyopathy.

## 2. Results

### 2.1. Clinical Report

The patient was the first offspring of healthy unrelated Italian parents of Caucasian ethnicity and was born at term by vaginal delivery with aid. She had a healthy brother. Family history was not contributory. Prenatal growth of the head occurred along the lower limit of the normal range. The karyotype from amniotic cells was 46,XX. Her birth weight was 3400 g (62nd centile), birth length 53 cm (98th centile), and occipitofrontal circumference (OFC) 32 cm (4th centile).

She sat alone at 7–8 months. The patient was referred to Neuropsychiatry Department at 16 months of age for psychomotor delay, congenital microcephaly and fine tremors at rest in the limbs. If supported, she could stand up on a widened base and walk a few steps. She had a severe language delay. At the clinical evaluation, weight was 9.5 kg (15th centile), height 75 cm (17th centile), and OFC 44 cm (6th centile); she exhibited bluish sclerae, posteriorly rotated ears and ligament hyperlaxity. At that time, brain MRI and EEG recordings were unremarkable, whereas lactic acid values were elevated in blood and cerebrospinal fluid. Muscle histology and activity of the muscle mitochondrial respiratory chain and of pyruvic dehydrogenase were normal.

**Figure 1:**
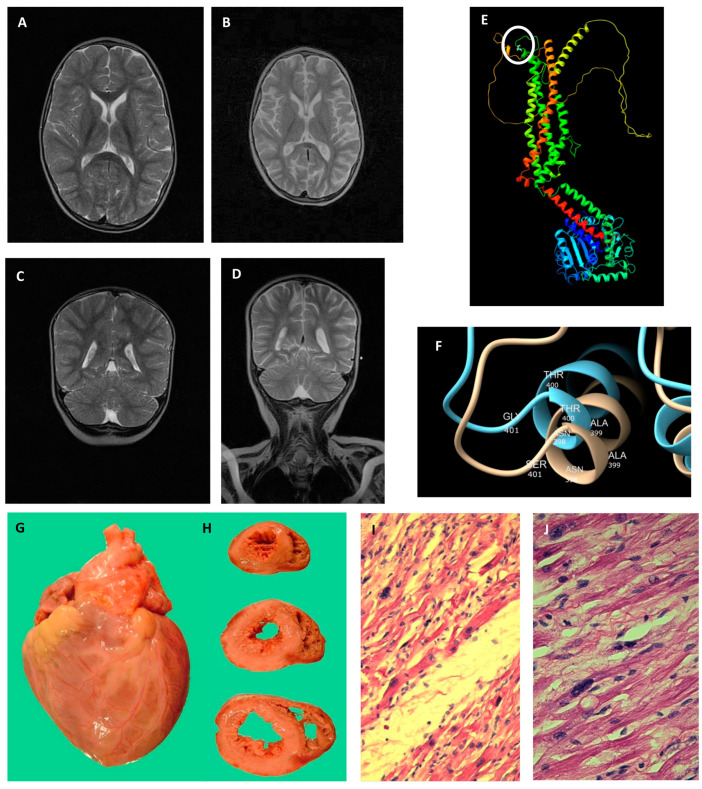
Brain MRI images showing rapidly progressive and diffuse cortical and subcortical atrophy, post-mortem presentation and histopathology of the heart, and structural consequences of the DRP1 p.(Gly401Ser) variant. (**A**) TSE T2 axial scans at the admission to the intensive care unit (at the age of 26 months) were comparable to those carried out at the age of 16 months. (**B**) TSE T2 axial scan on the same plane as the image shown in A was performed one month later (at 27 months). Brain MRI images show less cerebellar involvement compared to supratentorial structures, as shown in panel (**B**). (**C**) TSE T2 sagittal scan at the beginning of hospitalization (26 months) and (**D**) at the following month. In the protein 3D modeling for the *DNM1L*-wild-type (**E**), the white circle indicates the localization of 401 residues. The region of the Gly401Ser substitution (**F**): the wild-type protein is displayed in light blue, the mutated one in beige. Autoptic heart (**G**): weight was 110 g (proband’s weight 14.4 kg, at 61st centile). Coronal sections (**H**). Histology (20× panel (**I**), 40× panel (**J**)) with hematoxylin-eosin staining shows conspicuous myocellular hypertrophy and extensive interstitial fibrosis.

At 26 months, she suddenly developed therapy-refractory, multifocal myoclonic status epilepticus. Brain MRI performed at the beginning of hospitalization was comparable to that carried out previously (Figure 1A,C). She was intubated and placed on thiopental infusion; a burst suppression was obtained at the EEG with resolution of electrographic seizures by administering increasing dosages of thiopental. The patient became tetra-paretic and exhibited nystagmus and bursts of massive epileptic and non-epileptic myoclonus, with episodes of blood oxygen desaturation. For feeding, a nasogastric tube was applied, as well as Continuous Positive Airway Pressure (CPAP) to prevent sleep apnea. Brain MRI showed diffuse cortico-subcortical atrophy (Figure 1B) as well as mild cerebellar atrophy (Figure 1D). Ultrasound (US) of the heart was normal, whereas the US of the abdomen detected signs of mild hepatic steatosis with normal liver enzymes. Bone marrow was normal. She was released from the hospital after 2 months with Topamax 40 mg × 3/day and Gardenal 50 mg once a day for nasogastric tube, nasogastric tube feeding with nutrini 170 mL × 4/die, CPAP and indication for physiotherapy.

From the age of 29 months, she was fed with percutaneous endoscopic gastrostomy (PEG). At 32 months, she was diagnosed with hypertrophic cardiomyopathy, followed by epilepsia partialis continua after a urinary tract infection with fever. She was refractory to a number of antiepileptic drugs and did not tolerate a ketogenic diet. She died at the age of 36 months and 21 days from heart failure with right basal pneumonia.

An autopsy revealed brain spongiosis, gliosis, areas of neuronal loss and necrosis and congestive heart failure with severe hypertrophic cardiomyopathy (Figure 1G,H). The thickness of the left ventricular free wall, interventricular septum, and posterior right ventricular free wall was 12, 10 and 3 mm, respectively. Histology with hematoxylin eosin staining shows dysmorphic myocells with dysmorphic and hypertrophic nuclei (Figure 1I). At major resolution (Figure 1J), the myocells exhibit partially interrupted sections, a thin appearance with the structural anomaly of the contractile segments; some nuclei are conspicuously dysmorphic and hypertrophic. Moreover, there was evidence of severe endocardial fibrosis (Supplementary Figure S1A,B) and arteritis and periarteritis of the pulmonary arterial vessels (Supplementary Figure S1C). Neither cardiac deposits of glycogen nor mucopolysaccharides were identified.

### 2.2. Genetic Evaluation

The proband was wild-type for *POLG* pathogenic variants and for Copy Number Variants (CNVs) detectable at molecular karyotype (SNP-array). Southern blot of mtDNA extracted from the patient’s muscle biopsy did not show mtDNA deletions or depletion.

WES analysis of proband and parents carried out 12 years after the patient’s death allowed us to identify in the proband a de novo heterozygous *DNM1L* variant (NM_ 012062.5):c.1201G>A, p.(Gly401Ser) located in the MD. As c.1201G is the first nucleotide of exon 12, the G>A change may affect splicing, although alternative splicing was not predicted by the in silico tool. The missense variant was classified as likely pathogenic according to the ACGS/ACMG-AMP criteria PS2_strong, PM1 and PM2_moderate, PS3_supporting [8] (https://www.acgs.uk.com/media/11631/uk-practice-guidelines-for-variant-classification-v4-01-2020.pdf, accessed on 24 August 2024). Analysis of parental samples was undertaken as part of the trio WES pipeline, followed by targeted Sanger sequencing, which supported the de novo occurrence of the variant in the proband.

The p.(Gly401Ser) variant was absent from gnomAD (https://gnomad.broadinstitute.org/, accessed on 24 August 2024) and RCG variant browser (https://rgc-mcps.regeneron.com/, accessed on 3 January 2025) population databases. It occurred de novo in two unrelated patients described by Nolden et al. [9]. A comparison between these two patients and the one reported here is shown in Supplementary Table S1. The fission defect of mitochondria was demonstrated in one of the fibroblasts. Moreover, DRP1 levels and molecular weights in the patient’s cells were comparable to those of controls [9], making unlikely a remarkable effect of the variant on the splicing.

### 2.3. Structural Modelling of the p.(Gly401Ser) Variant

Based on the Alphafold model, the pathogenic variant lies at the end of alpha-helix n. 1, near a d-loop connecting the subsequent helix (Figure 1E), at the dimer interface. In Figure 1F, the structural impact of substituting glycine with serine can be observed: glycine, being the smallest amino acid, allows greater flexibility of the loop. In addition, the presence of serine, with its bulkier side chain and potential for hydrogen bonding, distorts this loop by altering the helix orientation. Moreover, computational analysis suggests that the substitution with a Ser in position 401 generates a destabilized protein (wild-type ROSIE score is 10,297.011, whereas the Gly401Ser score is 12,078.544), possibly hypo-functional or more prone to degradation.

### 2.4. Functional Studies in Yeast

**Figure 2:**
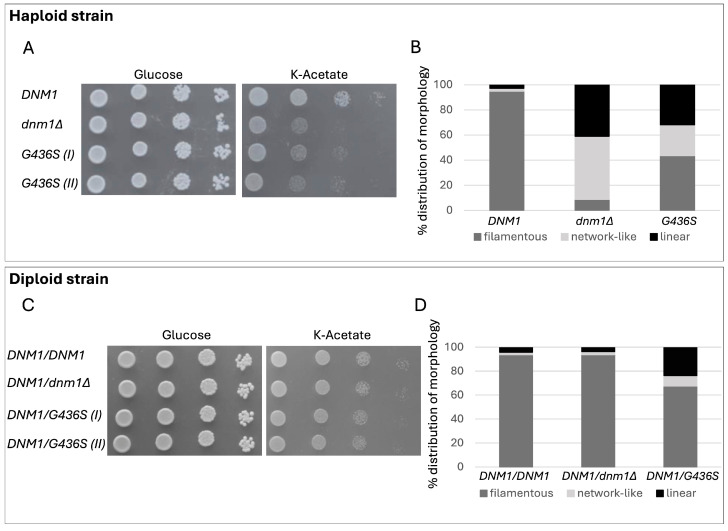
Functional analyses in the yeast *S. cerevisiae*. (**A**,**B**) Characterization of the haploid *dnm1Δ* strains harboring wild type allele (*DNM1*), the empty vector (*dnm1Δ*) or mutant allele *dnm1^G436S^* (G436S). (**A**) Oxidative growth: the strains were serially diluted and spotted on SC agar plates supplemented with the fermentable carbon source glucose (2%) or the non-fermentable carbon source K-acetate (2%) and incubated at 37 °C. (**B**) Mitochondrial morphology. For each strain, the percentage of the following morphotypes is reported: filamentous (in dark grey), network-like (in light grey) and linear (in black). (**C**,**D**) Characterization of the diploid *DNM1*/*dnm1Δ* strain harboring wild-type allele (*DNM1*/*DNM1*) mutant allele *dnm1^G436S^* (*DNM1*/G436S) or the empty vector (*DNM1*/*dnm1Δ*). (**C**) Oxidative growth: the strains were serially diluted and spotted on SC agar plates supplemented with the fermentable carbon source glucose (2%) or the non-fermentable carbon source K-acetate (2%) and incubated at 37 °C. (**D**) Mitochondrial morphology. For each strain, the percentage of the following morphotypes is reported: filamentous (in dark grey), network-like (in light grey) and linear (in black).

The yeast *S. cerevisiae* was used as a model to study the functional effect of the p.(Gly401Ser) variant identified in the patient, as the patient’s derived cells and tissues were unavailable, having died 12 years before carrying out the WES. A homologous complementation approach exploited *DNM1*, the yeast ortholog of human *DNM1L*. The corresponding codon was mutagenized, creating the mutant allele *dnm1^G436S^*. The wild-type allele *DNM1*, the mutant allele *dnm1^G436S^*, or the empty vector (EV), were introduced in the yeast haploid *dnm1Δ* null mutant, thus obtaining the strains *dnm1Δ*/*DNM1*, *dnm1Δ*/*dnm1^G436S^* and *dnm1Δ*/EV, respectively. Oxidative growth was determined through spot assay using a medium containing acetate as a non-fermentable carbon source at 37 °C. The growth of the strain carrying *dnm1^G436S^* was strongly reduced (Figure 2A). Likewise, the oxygen consumption rate was severely reduced in the mutant strain *dnm1^G436S^* (61% residual rate relative to the *DNM1* wild-type strain), similar to the null mutant strain (55%) (Supplementary Figure S2A). Together, the data show that the yeast Gly436Ser variant, equivalent to the human mutation p.(Gly401Ser), is deleterious in yeast, supporting its pathogenicity in humans.

Pathogenic variants in *DNM1* [1,10,11] and in several nuclear genes encoding proteins involved in mitochondrial dynamics [12,13,14] affect mtDNA stability in yeast. We showed that the lack of *DNM1* and the expression of mutant *dnm1^G436S^* resulted in a significant increase in *petite* colonies, a hallmark of mtDNA instability in yeast (approximately 41% and 46%, respectively, whereas wild-type *DNM1* had 5.8% of *petites*) (Supplementary Figure S2B).

The absence of the Dnm1 protein has a detrimental effect on the fission process and determines a network-like or linear mitochondrial morphology [10,15]. We transformed the haploid strains with a plasmid encoding a mitochondrial-targeted GFP to allow visualization of the mitochondrial morphology [1] (Supplementary Figure S4A). Most of the wild-type cells (*DNM1*) showed a filamentous network (95%), typical of strains with a well-functioning fusion-fission process. The null mutant strain showed predominantly network-like (50%) or linear (41%) morphotypes. The strain *dnm1^G436S^* showed an intermediate behavior, showing filamentous (43%), network-like (25%), or linear (32%) morphotypes (Figure 2B).

To further confirm the fission defects, cells were treated with sodium azide, which determines the fragmentation of mitochondria when the mitochondrial fission machinery is properly functioning (Supplementary Figure S4B). The mitochondrial network of the strain carrying wild-type *DNM1* became almost completely fragmented (92%), whereas in the null *DNM1* strain, only 26% of the cells showed fragmented mitochondria. The strain *dnm1^G436S^* presented an intermediate phenotype, as 63% of the mitochondrial network became fragmented (Supplementary Figure S2C).

Furthermore, we studied the dominant/recessive behavior of Gly436Ser by introducing the mutant allele *dnm1^G436S^* in the diploid *DNM1*/*dnm1Δ* strain thus obtaining *DNM1*/*dnm1^G436S^*; in addition, the strains *DNM1*/*DNM1* and *DNM1*/*dnm1Δ* were constructed as controls, by transforming the same strain with *DNM1* allele or the empty vector, respectively. Oxidative growth of the *DNM1*/*DNM1* and *DNM1*/*dnm1Δ* was similar, whilst the strain *DNM1*/*dnm1^G436S^* showed a moderate growth reduction on the non-fermentable carbon source K-acetate (Figure 2C). The respiratory activity was also impaired, associated with a significant reduction of oxygen consumption (90% residual rate relative to the wild-type; Supplementary Figure S3A), indicating that the pathogenic variant Gly436Ser behaves as a dominant allele. In addition, the *petite* frequency of *DNM1*/*dnm1^G436S^* was significantly higher than that of *DNM1*/*DNM1* and *DNM1*/*dnm1Δ* (Supplementary Figure S3B). Morphologically, the *DNM1*/*DNM1* and *DNM1*/*dnm1Δ* strains showed mostly filamentous networks (around 94% in both); the strain *DNM1*/*dnm1^G436S^* showed filamentous network (67%), but also network-like (9%) or linear (24%) morphotypes (Figure 2D). Upon sodium azide treatment, in the *DNM1*/*DNM1* and *DNM1*/*dnm1Δ* strains, around 78% of the mitochondrial network became fragmented; in the *DNM1*/*dnm1^G436S^* only 58% of the cells presented mitochondrial fragmentation (Supplementary Figure S3C). Altogether, the results support the dominance of the human Gly401Ser variant. The phenotype of the *DNM1*/*dnm1^G436S^* strain was consistently worse than that of the *DNM1*/*dnm1Δ* hemizygous strain, indicating that the dominance is not due to haploinsufficiency but to a dominant-negative effect, as previously reported for other pathogenic variants [1,11,16].

### 2.5. Literature Review

**Figure 3:**
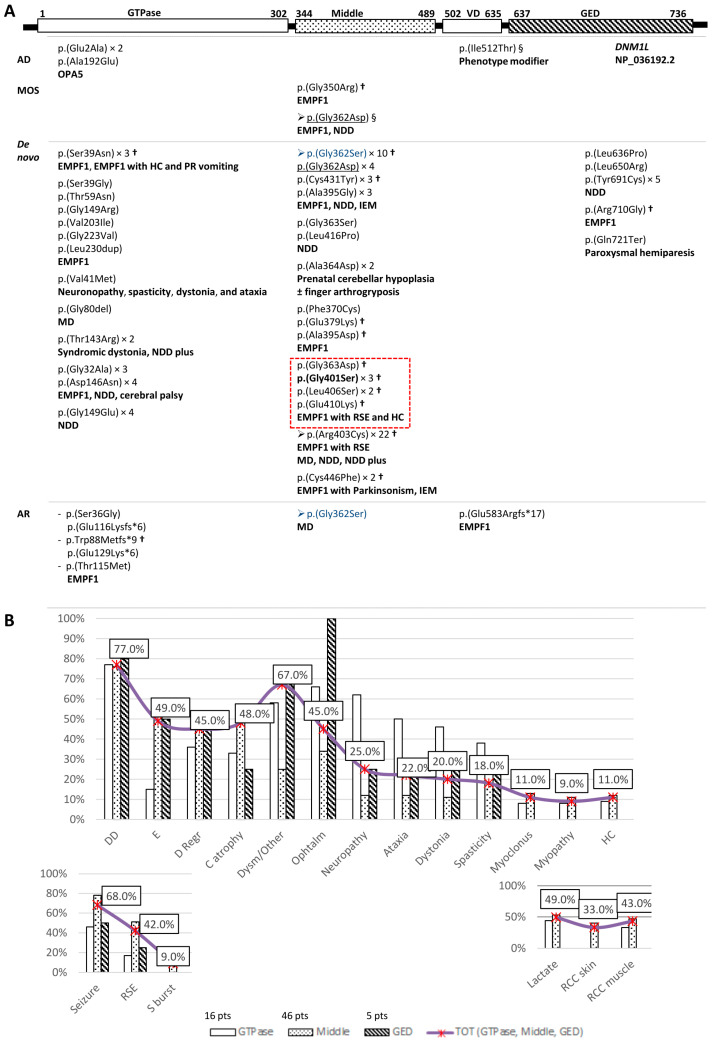
Genotype-phenotype correlation of *DNM1L*-related neurological disorders. (**A**) Pathogenic variants were distributed at each protein domain and according to their inheritance. Phenotypes due to each pathogenic variant are shown. EMPF1, encephalopathy due to defective mitochondrial and peroxisomal fission 1; OPA5, optic atrophy 5; HC, hypertrophic cardiomyopathy; MD, mitochondrial disease; NDD, neurodevelopmental disorder; RSE, Refractory Status Epilepticus; PR, paroxysmal refractory; IEM, inborn errors of metabolism; VD, variable domain; GED, GTPase effector domain; AD, autosomal dominant; AR, autosomal recessive; MOS, mosaic; §, in cis identified variants; × n, number of times a pathogenic variant has been described in unrelated patients (recurrent pathogenic variant); arrow, mutational hot spot; pathogenic variants with different inheritance patterns were underlined or colored in blue; in bold, the pathogenic variant identified in the present patient; †, variants for which lethality has been reported; in the dashed red box those pathogenic variants associated with epileptic encephalocardiomyopathy. Patient with isolated paroxysmal hemiparesis was reported by Zhang et al. [43]. (**B**) Distribution of the clinical features shown by *DNM1L* heterozygotes, including the present case, across the three protein domains, GTPase (white rectangles), MD (rectangles with dots) and GED (rectangles with diagonals). DD, developmental delay; E, encephalopathy; D Regr, Developmental Regression; C atrophy, cerebral atrophy; dysm/other, dysmyelination/Other brain RMN abnormalities; ophtalm, the presence of optic nerve atrophy and/or Poor visual fixation and/or nystagmus, and/or strabismus; RSE, refractory status epilepticus; S burst, suppression burst; Lactate, increased serum and/or CSF lactate; RCC skin, reduction of the activity of the respiratory chain complexes in skin; RCC muscle, reduction of the activity of the respiratory chain complexes in muscle. Percentages refer to the number of patients showing the clinical feature independently from the genotype subgroup. Among the numerous neurological features placed in order of decreasing frequency, those with impairment of higher brain functions have been grouped on the left. The biochemical items and those concerning epilepsy have been set apart.

Clinical and genetic data from all the published patients affected by a *DNM1L*-related disorder are summarized in Figure 3. Ninety-seven unrelated probands (de novo occurrence of the pathogenic variant except in one homozygous proband), three couples of siblings (autosomal recessive inheritance) and one couple of half-siblings (autosomal dominant inheritance with low-level mosaicism in the mother’s blood), for 44 different *DNM1L* pathogenic variants, have been reported (Figure 3A). The median age of onset and/or first evaluation was 24 months (from birth to the eleventh year of life). The median age of the last follow-up was 9.5 years (from the age of 8 days to 21.5 years/adulthood). Lethality was 27%, 29.5% and 20% for patients carrying a pathogenic variant in the GTPase, middle and GED domains, respectively. The total lethality percentage was 28%. Death occurs from the eighth day to the seventeenth year of life.

Among 111 heterozygous patients, 35 belonged to cohorts classified as: neurodevelopmental disorders (NDDs) [17,18,19,20,21,22,23,24,25,26,27], autism [28], refractory epilepsy [29], syndromic movement disorders [30], cerebral palsy [31], rare disorders [32,33,34,35], inborn errors of metabolism [36,37], mitochondrial disorders [38,39], Leigh syndrome [40], or prenatal cases [41,42].

Figure 3B displays the clinical data of 67 heterozygous patients, including the proband described here and 9 out of the 35 patients from the cohorts. Those affected by OPA5 (16) were excluded.

As shown in Figure 3B, impairment in higher brain functions played a major role in determining the phenotype, with a difference among the DRP1 domains: encephalopathy and developmental regression were prevalent in patients with a pathogenic variant in the middle and GED domains, whereas peripheral neuropathy, ataxia, dystonia and spasticity were more frequent in pathogenic variants of the GTPase domain. There was no difference in developmental delay among the three sub-groups. Interestingly, seizures, refractory status epilepticus (RSE) and development of cerebral atrophy were more frequent in patients with a pathogenic variant in the MD. The suppression burst pattern at EEG occurred only in the MD sub-group. Recurrent brain abnormalities included corpus callosum hypoplasia/atrophy, T2 hyperintensities in thalami, and cerebellar hypoplasia. Interestingly, the brain MRI of the two patients with p.(Leu406Ser) was typical of Leigh syndrome [40,44]. Not all the patients with *DNM1L* pathogenic variants exhibited lactic acidosis and/or respiratory chain deficiencies in muscle and/or skin fibroblasts (Figure 3B).

Prenatal phenotype (found in overall 16% of the patients) included oligohydramnios [45], fetal growth restriction (FGR) [9,11] (Supplementary Table S1), FGR and hydrocephalus [45], cerebellar hypoplasia [41], decreased fetal movement [46], and head growth at the lower limit of the normal (present case) (Supplementary Table S1). Microcephaly, failure to thrive, and poor feeding accounted for 23%, 27% and 15% of the patients, respectively, and none of them were in the group of the GED domain.

As a whole, dysmorphisms were reported in 25% of the patients. There was no facial gestalt associated with *DNM1L*-related neurological disorders, although recurrent craniofacial dysmorphisms included triangular/long faces, deep-set eyes, pointed chin/micrognathia, and high (arched) palate.

## 3. Discussion

*DNM1L*-related phenotype ranges from non-syndromic optic atrophy [5] to infantile/early childhood onset lethal encephalopathy [2,9,11,44,45,46,47,48,49] through an ‘intermediate EMPF1’. These ‘intermediate’ patients could exhibit a later lethal [9,42,50,51,52] or static encephalopathy (for instance, Lhuissier et al. [53]) with [54,55,56,57] or without several neurological features (Figure 3B) and/or epilepsy, or be indistinguishable from a ‘Neurodevelopmental disorder’ (NDD), such as for patient 5 in Whitley et al. [45] (Figure 3A). Indeed, a consistent number of patients (27%) belonged to the cohort of NDDs/autism [17,18,19,20,21,22,23,24,25,26,27,28]. Moreover, *DNM1L* pathogenic variants have been identified in a cohort of patients with movement disorders [30] and cerebral palsy [31]. The onset of status epilepticus, followed by neurological deterioration and brain atrophy, and subsequent fatal outcome, could be insidious on a background of developmental and/or speech delay/learning disability/intellectual disability [50,58,59] or even normal development [29,60,61], as described in patients affected by the p.(Arg403Cys) [29,50,58,59,60,61].

Here, we report on a baby girl with developmental delay who died in her third year of heart failure due to hypertrophic cardiomyopathy after developing a therapy-refractory myoclonic status epilepticus and neurological deterioration with brain atrophy and refractory epilepsy. At the time of her death (2011), genetic analyses did not reveal any cause.

Likewise, lactic acid was elevated in only half of the patients with a pathogenic variant in *DNM1L*, including our own patient (Figure 3B). Lactic acidosis was, in fact, her initial sign. Her subsequent clinical history, i.e., progressive neurological deterioration with seizures (status epilepticus and epilepsia partialis continua), is more clearly oriented towards a *DNM1L* pathogenic variant (Figure 3B). These critical conditions should prompt the analysis of *MT-TL1* (m.3243A>G), *POLG*—carried out when she was alive—and *DNM1L* variants [39]. By WES-trio, she was found to be heterozygous for the p.(Gly401Ser).

As for the hypertrophic cardiomyopathy (HC), which was the cause of death of our patient, it was reported in one of the two patients with the p.(Gly401Ser) [9] (Supplementary Table S1), in other three patients with a *DNM1L* pathogenic variant in the MD [44,49,62] and in one patient with a pathogenic variant in the GTPase domain [52] (Figure 3A). Interestingly, a mouse model of dilated cardiomyopathy is heterozygous for the *DNM1L* pathogenic variant in the MD [63].

We also found that some pathogenic variants of *DNM1L* MD (framed in Figure 3A), including the p.(Gly401Ser), were associated with encephalocardiomyopathy characterized by RSE. Therefore, management and follow-up of patients with *DNM1L*-associated mitochondrial encephalopathy must involve cardiac screening and monitoring.

In adulthood, HC (prevalence of 1:500) is typically caused by pathogenic variants in sarcomere genes, whereas pediatric HC is most frequently associated with Noonan syndrome (OMIM # 163950) and with IEM, including MDs [64]. Cardiomyopathy is estimated to occur in 20–40% of children with MDs and might impact severity (mortality of 71% vs. 26% without cardiac involvement). HC is the most common type; however, mitochondrial cardiomyopathies might also present as dilated, restrictive, left ventricular non-compaction, and histiocytoid cardiomyopathies, with congenital arrhythmias and congenital heart defects being part of the MD clinical spectrum. HC was reported to be associated with about 90 MD genes out of 100 with a cardiac phenotype [65]; these genes are distributed in each of the pathways in which the mitochondrial genes have been organized (https://genomit.eu/work-packages/integrated-diagnostics/disease-genes/index.html, accessed on 29 December 2024). Based on this extensive literature review, *DNM1L* must be included among the mitochondrial homeostasis genes.

Optic nerve atrophy was rarely (13%) described in the *DNM1L* pathogenic variants associated with the EMPF1 spectrum. However, ophthalmological involvement –including optic nerve atrophy, poor visual fixation, nystagmus, and/or strabismus– was overall 45% of the EMPF1 heterozygous patients.

Identification of *DNM1L* pathogenic variants in the cohorts of patients affected by NDDs and autism prompted us to account for behavioral abnormalities during the review of clinical reports. We found reports of early onset self-injurious behavior (biting fingers and self-inflicted mouth ulcers/severe damage of the tongue) [11,51,66], self-mutilation (Decipher Patient’s ID 280556), ADHD, autistic features, hyperventilation [45], inattention and intermittent aggressive behavior [60], anxiety, perseveration, night terrors, parasomnias, and scary dreams [50], increased startle response [49], and behavioral changes [67].

From a genetic point of view, only two *DNM1L* variants in the GTPase domain have been associated in three unrelated families with isolated optic atrophy reported as OPA5 mutants (Figure 3A). Autosomal dominant inheritance has also been described for EMPF1 due to two pathogenic variants of the MD, but the carrier mothers had low-level mosaicism in blood, i.e., 5% for the p.(Gly362Asp) [11] and 8% for the p.(Gly350Arg) [46] (Figure 3A).

Five out of the seven different pathogenic autosomal recessive variants fall in the GTPase domain (Figure 3A). The most severe family in the literature was composed of two compound heterozygous sibs, who both died at birth of ventilatory insufficiency. They carried two early truncating pathogenic variants, p.(Trp88Metfs*9) and p.(Glu129Lys*6) [2]. However, early lethality has also been reported for monoallelic pathogenic variants [9,11,44,45,46,47,48,49] (Figure 3A).

A less severe, intermediate EMPF1 phenotype was found in consanguineous patients homozygous for the p.(Thr115Met) [3], or compound heterozygous patient for the p.(Ser36Gly) and the early truncating p.(Glu116Lysfs*6) [1]. Interestingly, a similar ‘intermediate EMPF1 phenotype’ occurred also in a single patient homozygous for the late truncating p.(Glu583Argfs*17) located in the VD domain [68], suggesting some residual activity of the mutant protein.

*DNM1L* pathogenic variants are almost all missense, and invariably so in those located in the MD. Some of them (Figure 3A) were recurrent in unrelated patients, with two mutational hotspots in the MD: the p.(Arg403Cys), found in 22 probands [11,24,25,28,29,33,35,39,45,50,58,59,60,61,67,69,70]; the p.(Gly362Ser), identified in 10 probands [11,22,23,24,28,34,51,66] (Decipher Patient ID 280,556 and ID 389904), and the p.(Gly362Asp) in 4 probands and a couple of half-sibs [11,18,21,37,71] (Figure 3B). Interestingly, the p.(Gly362Ser) was reported by Wu et al. [39] in homozygosity, suggesting the mosaicism in one of the parents, as also reported for the p.(Gly350Arg) and p.(Gly362Asp), and the de novo occurrence in the other allele. Furthermore, the p.(Ile512Thr), in the VD domain, has been proven to be a phenotype modifier [11,72], being found in cis with the p.(Gly362Asp) [11].

Evidence of low-level mosaicism for some *DNM1L* pathogenic variants (p.Gly350Arg, p.Gly362Asp and hypothesized for Gly362Ser) and of germline mosaicism (p.Gly362Asp) indicate prenatal diagnosis in pregnancies of couples with a child in the EMPF1 spectrum due to an apparently de novo *DNM1L* pathogenic variant.

As for our pathogenic variant p.(Gly401Ser), we showed that the equivalent variant in yeast is associated with instability of mtDNA (increased *petite* colonies), although Southern-blot analysis of the muscle mtDNA in our patient failed to show large-scale rearrangements of mtDNA or depletion. Notably, in the diploid system, the heteroallelic yeast strain carrying the p.(Gly436Ser) variant was even more affected than both the hemizygous *DNM1*/*dnm1Δ* and the wild-type strains, suggesting a dominant negative effect of the p.(Gly401Ser) variant.

Further insight into the pathogenicity of the variant was gained through observation of the mitochondrial network in yeast. Compared to the wild-type, the haploid *dnm1^G436S^* mutant strain exhibited a higher percentage of linear and net-work-like morphotypes, a pattern also observed in the diploid heteroallelic mutant strain. Interestingly, the *DNM1/dnm1Δ* diploid strain displayed a morphotype pattern very similar to that of the wild-type, again indicating a dominant negative effect of the p.(Gly401Ser) variant. Results from the fragmentation of the mitochondrial network with sodium azide test were similar to these, confirming the fission defect and the underlying pathomechanism. These results fit with that reported by Nolden et al. [9]. The authors, after demonstrating the fission defect of mitochondria in derived fibroblasts of a patient affected by the p.(Gly401Ser), observed that monomeric DRP1 levels in the patient’s cells were comparable to those of age-matched controls, suggesting that the mutant protein may act in a dominant-negative fashion, overriding the effect of the wild-type allele. Indeed, DRP1 exists in a dynamic equilibrium between dimers and tetramers, which ultimately leads to higher-order assembly [73]. Through SEC-MALS (size-exclusion chromatography with multi-angle laser light scattering) analysis, Nolden et al. [9] provided strong evidence that the p.(Gly401Ser), like other pathogenic variants in this middle region [73], impairs DRP1 ability to self-assemble by altering the exchange rate between dimers and tetramers, potentially disrupting the assembly-dependent stimulation of the GTPase activity, which is critical for mediating mitochondrial fission.

When looking at the protein structure, residue Gly401 serves as a termination residue for α-helix 1 in the stalk domain and is located at the dimer interface, where it is predicted to play a role in the self-interaction of DRP1 monomers (PDB Entry - 5WP9, available at https://doi.org/10.2210/pdb5wp9/pdb, accessed on 29 December 2024). Substitution of glycine to a polar serine would induce steric clashes with neighboring residues and likely result in an energetically unfavorable conformation that slightly destabilizes the helix, disturbing intermolecular interaction and self-assembly of DRP1.

Altogether the functional studies in yeast not only validated the pathogenicity of the *DNM1L* (NM_012062.5):c.1201G>A, p.(Gly401Ser) but also demonstrated that it acts through a dominant negative effect over the wild-type allele, as already reported for other pathogenic variants located in the MD of DRP1 [11,45,46,47,48,60,73] as well as in GTPase domain [1,45,74].

Yeast is a valid model to demonstrate the pathogenicity of variants in nuclear genes encoding for mitochondrial proteins identified in deceased patients for whom there are no cells if they involve codons coding the same amino acid. Moreover, analyses with diploid yeast strains should also be considered when a novel *DNM1L* variant is identified since a hypomorphic allele [11], and bi-allelic inheritance [1,2,3,39,68] have been reported for the gene.

## 4. Materials and Methods

### 4.1. Subjects

The baby girl belongs to a retrospective cohort of patients who were re-analyzed as suspected of suffering from a mitochondrial disease. Parents were contacted for blood sampling if their DNA had not previously been collected. Written informed consents were obtained from the parents of the patient to participate in the study.

### 4.2. Brain Imaging

Brain Magnetic resonance imaging (MRI) examinations were performed under sedation in 2010 on a Philips Panorama 0.6 T scanner (Philips, The Netherlands). The scans consisted of TSE T2 images.

### 4.3. Mutational Analysis

Genomic DNA was extracted from the patient’s muscle biopsy and from peripheral blood samples of parents using standard procedures. We used 250 ng DNA as a template for the construction of a paired-end library, according to the Illumina DNA Prep with Enrichment protocol (Illumina, San Diego, CA, USA) and with xGen IDT probes targeting a panel of 300 genes associated with mitochondrial pathology. Libraries were sequenced on a MiSeq instrument (Illumina). The sequencing reads were aligned to the NCBI human reference genome (GRCh37/hg19) using the Burrows–Wheeler Aligner (BWA version 0.7.17). Single nucleotide variants (SNVs) and small insertions/deletions (INDELs) calling were performed using GATK4.1.

Variant interpreter software (https://variantinterpreter.euc1.vi.basespace.illumina.com, accessed on 2 January 2023) was used for variants annotation and filtering; variants with a minor allele frequency (MAF) ≥ 1% in the 1000 Genomes Project (http://www.1000genomes.org, accessed on 2 January 2023), dbSNP (https://www.ncbi.nlm.nih.gov/snp/, accessed on 2 January 2023), and GnomAD databases (https://gnomad.broadinstitute.org, accessed on 2 January 2023) were discharged. The subsequent analysis focused on the exonic regions and splicing sites as well as the proband’s phenotype.

### 4.4. Structural Analysis

The protein models were prepared using AlphaFold (version 2), an advanced tool for protein structure prediction, followed by relaxation using the Rosetta Online Server that Includes Everyone (R.O.S.I.E.). Initially, a structural model of the wild-type human protein was generated using AlphaFold. This model served as the template for further mutagenesis. Specifically, the glycine residue at position 401 (Gly401) was substituted with serine (Ser) to create a mutant model reflecting the desired sequence alteration.

Both the wild-type and the mutant models were subjected to further processing to ensure structural accuracy and energy minimization. To achieve this, the highest-confidence (top-score) models produced by AlphaFold were relaxed using the ‘Re-lax’ application available on the R.O.S.I.E. platform (Rosetta server version 3.14). This process optimized side-chain conformations and minimized the overall free energy of the structures, resulting in more stable and realistic models.

After relaxation, the ‘Score’ application within R.O.S.I.E. was used to assess the energetic properties of each model. The scoring analysis provided insight into the relative stability of the wild-type and mutant structures, helping to quantify the impact of the Gly401Ser substitution on protein stability and conformation.

### 4.5. Yeast Strains and Growth Conditions

The yeast strains used in this work are W303-1B dnm1Δ (MATα, leu2-3, trp1-1, can1-100, ura3-1, ade2-1, his3-11, dnm1::KanMX4) and W303 DNM1/dnm1Δ (MATa/MATα, leu2-3/leu2-3, trp1-1/trp1-1, can1-100/can1-100, ura3-1/ura3-1, ade2-1/ade2-1, his3-11/his3-11, DNM1/dnm1::KanMX4) [1]. The dnm1^G436S^ mutant allele was generated with the PCR QuikChange technique using DNM1 cloned in the pFL38 plasmid as a template. All the plasmids and the corresponding empty vector were used to transform the haploid W303-1B dnm1Δ or the diploid W303 DNM1/dnm1Δ yeast strains, using the lithium acetate method [75] after growth in YPAD medium (1% yeast extract, 2% peptone, 75 mg/L adenine, 2% glucose (Formedium™, Norfolk, UK)). For all the experiments, except for transformation, cells were grown in a liquid SC medium (0.69% YNB without amino acids, 0.1% Kaiser Drop out mix without uracil and/or tryptophan necessary for transformants selection (Formedium™, UK), under constant shaking at 28 °C or 37 °C, or in solid SC medium containing 2% agar for solidification (Formedium™, UK). Media were supplemented with various carbon sources (Carlo Erba Reagents, Cornaredo, Italy), as indicated in the results and figures. For growth analyses, the strains were serially diluted, spotted, and grown at 37 °C on solid SC medium plates supplemented with 2% glucose or 2% sodium acetate.

### 4.6. Yeast Analyses

Mitochondrial respiratory activity was evaluated by measuring the oxygen consumption rate using a Clark-type oxygen electrode (Oxygraph System Hansatech Instruments England, Norfolk, UK) at 30 °C with 1 mL of air-saturated respiration buffer (0.1 M phthalate–KOH pH 5.0, 0.5% glucose) from yeast cells cultured in liquid SC without uracil medium supplemented with 0.6% glucose at 37 °C until exhaustion (approximately 16 h).

*Petite* frequency was determined in solid medium as previously described [76] in at least four different clones for each strain.

To determine mitochondrial morphology, the strains were transformed with a mitochondrial-targeted GFP (mtGFP) on pYX232 plasmid [77]. The mtGFP-transformed cells were grown at 37 °C under constant shaking in 2 mL of SC without uracil and tryptophan medium supplemented with 0.6% glucose. Once diluted to 1 × 10^7^ cells/mL, the cells were observed with a Nikon Eclipse E600 epifluorescence microscope (Nikon Corporation, Tokyo, Japan) using a Nikon 100X Plan Fluor Oil objective and a Nikon Green Excitation Filter Block G-2A. Images were acquired and elaborated with the NIS-Elements F 4.00.00 microscope. Mitochondria were manually counted and belong to three different morphotypes as previously described [1]: (a) filamentous with long and branched mitochondria, (b) linear with long, unbranched mitochondria, and (c) network-like, in which filamentous mitochondria are fused and form net-like structures. A total of 200 single cells deriving from two independent clones were analyzed for each strain. For sodium azide treatment, cells were grown as above and treated for 45 min with 1.5 mM sodium azide, washed twice with water, diluted at 1 × 10^7^ cells/mL, and visualized. An example of mitochondrial network/fragmentation visualization is reported in Supplementary Figure S4.

### 4.7. Literature Review Selection

We analyzed the clinical reports of manuscripts indexed in PubMed (accessed on 26 August 2024) of patients with a *DNM1L* pathogenic/likely pathogenic variant, filling a database with the available clinical information.

We also included patients from the large cohorts of Developmental Disorders diagnosed by means of (trio) exome sequencing or by re-analysis of clinical exome sequencing data. For this purpose, we also used HGMD^®^ Professional (https://digitalinsights.qiagen.com/), ClinVar (https://www.ncbi.nlm.nih.gov/clinvar/) and Decipher (https://www.deciphergenomics.org) databases (accessed on 26 August 2024).

Microcephaly was accounted for when the measure of OFC was at least -2 SD compared to the height unless it was reported without auxological measurements. ‘Super refractory *status epilepticus*’ was accounted for as ‘refractory *status epilepticus*’ (RSE).

## Data Availability

Data are contained within the article or Supplementary Materials.

## Supplementary Materials

The supporting information can be downloaded at: https://www.mdpi.com/article/10.3390/ijms26020846/s1.
