# Supplementary material for: De Novo DNM1L Pathogenic Variant Associated with Lethal Encephalocardiomyopathy—Case Report and Literature Review
Source: Int J Mol Sci. 2025 Jan 20;26(2):846. doi: 10.3390/ijms26020846 (PMC11765995; doi:10.3390/ijms26020846)
Supplement: Supplementary file 1 [file ijms-26-00846-s001.zip › ijms-3381693-supplementary.pdf]

Supplementary Table S1. Clinical features of EMPF1 patients with the p.(Gly401Ser).

The patient here described exhibited the symptoms of both the infants described by Nolden et al (2022). She had cardiomyopathy and elevated lactate, as in P1, and epileptic encephalopathy, as in P5, but she differentiated compared to P1 and P5, because she developed severe neurological deterioration with rapidly progressive and diffuse cortical and subcortical atrophy due to RSE.

Patient, Gender	Age at onset (months, m)	Clinical course	Epileptic encephalopathy	SE	Developmental regression/ Neurological deterioration	Neurological examination	Brain MRI	Lactate	Cardiomyopathy	Other
P1 (Nolden 2022), F	8	† 10m	-	-	+ (sudden, in feeding and breathing)	DD, hypotonia	- (mild thinning of CC)	↑	HC	Prenatal FGR, postnatal growth at lower limit
P5 (Nolden 2022), M	33	Alive 3y	+ (early onset)	+	+	Nystagmus, DD, hypotonia, dyskinesia	-	-	-	-
Present study, F	16	† 36m	+	+, RSE	+	Nystagmus, DD, tetraparesis	Rapidly progressive and diffuse cortical and subcortical atrophy	↑	HC	Head growth at the lower limit since prenatal period, postnatal growth at lower limit

DD, developmental delay; HC, hypertrophic cardiomyopathy; SE, status epilepticus, RSE, refractory status epilepticus; CC, corpus callosum; FGR, fetal growth restriction; +, present; -, normal/absent; †, deceased; m, months; y, years; F, female; M, male

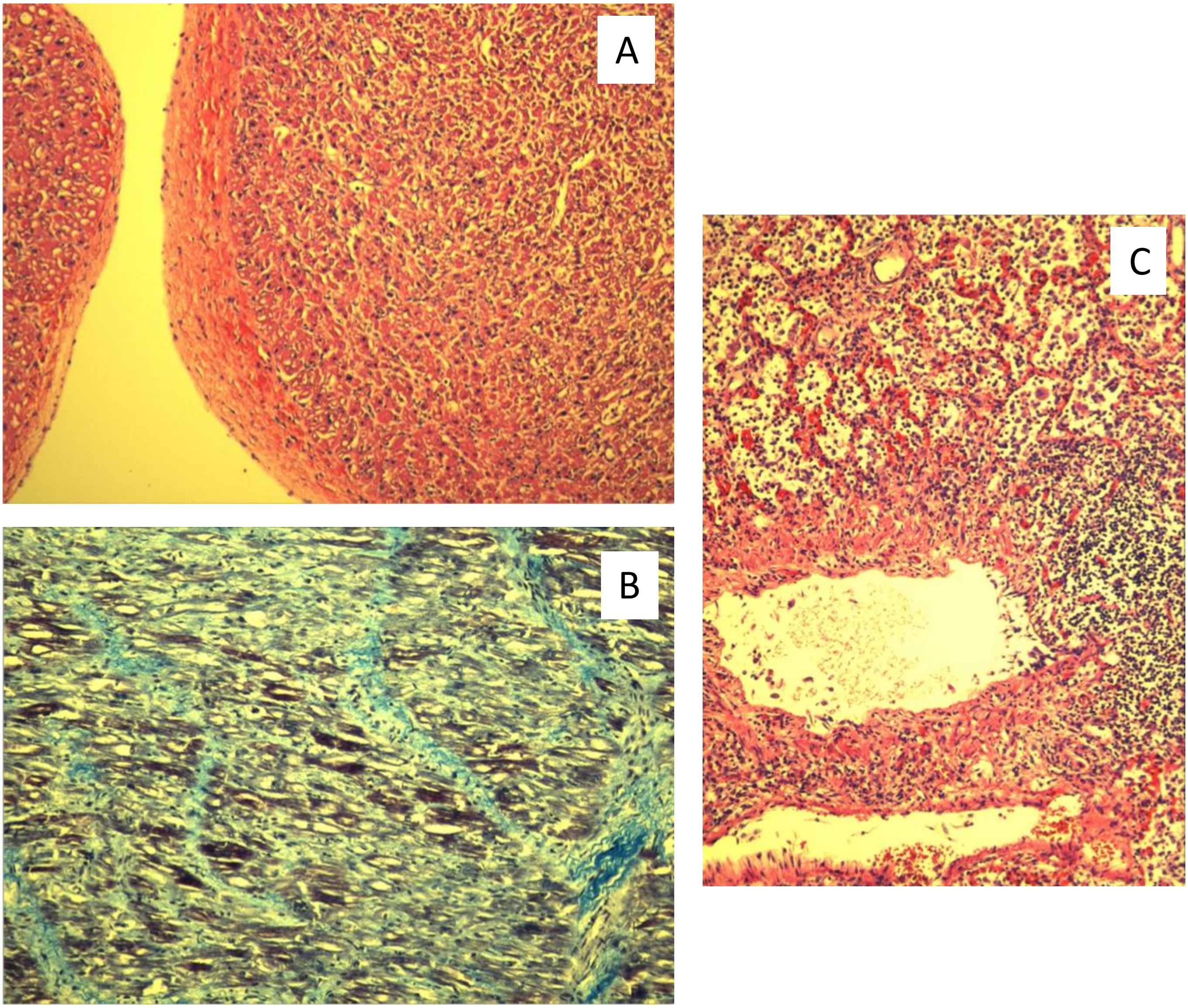

Supplementary Figure S 1. Heart's histopathological pictures showing significantly increased subendocardial thickness (2.5x panel A) and interstitial fibrosis (10x Azan-Mallory staining panel B). Lung's histopathological picture depicting intense congestion and inflammatory infiltrate around a vascular wall (2.5x panel C).

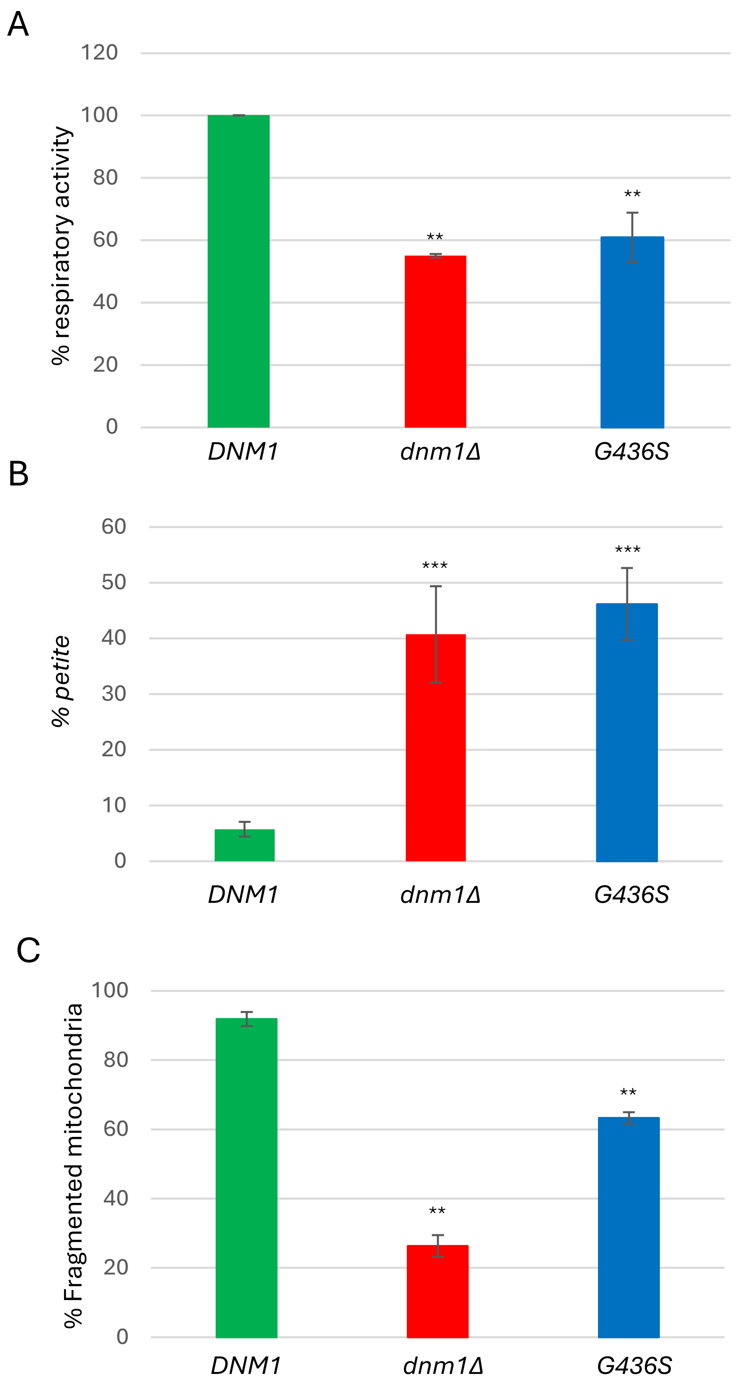

Supplementary Figure S2 **Additional analyses in the haploid yeast *S. cerevisiae***
A. Oxygen consumption rate of the haploid *dnm1Δ* strain harboring wild-type *DNM1* (green), the empty vector (red) or the mutant allele$dnm1^{G436S}$ (blue). Cells were grown at 37 °C in SC medium without uracil supplemented with 0.6% glucose till exhaustion. Values are represented as the mean of at least four values ±SD. **B**. *Petite* frequency (±SD) of the same strains as in (A) at 37°C. **C**. Mitochondrial morphology of same strains as in (A) after treatment with sodium azide. For each strain, the percentage of cells (± SD) showing fragmented mitochondria is reported. Statistical analysis was performed using a one-way ANOVA followed by Bonferroni's post-hoc test: ** $p < 0.01$; *** $p < 0.001$

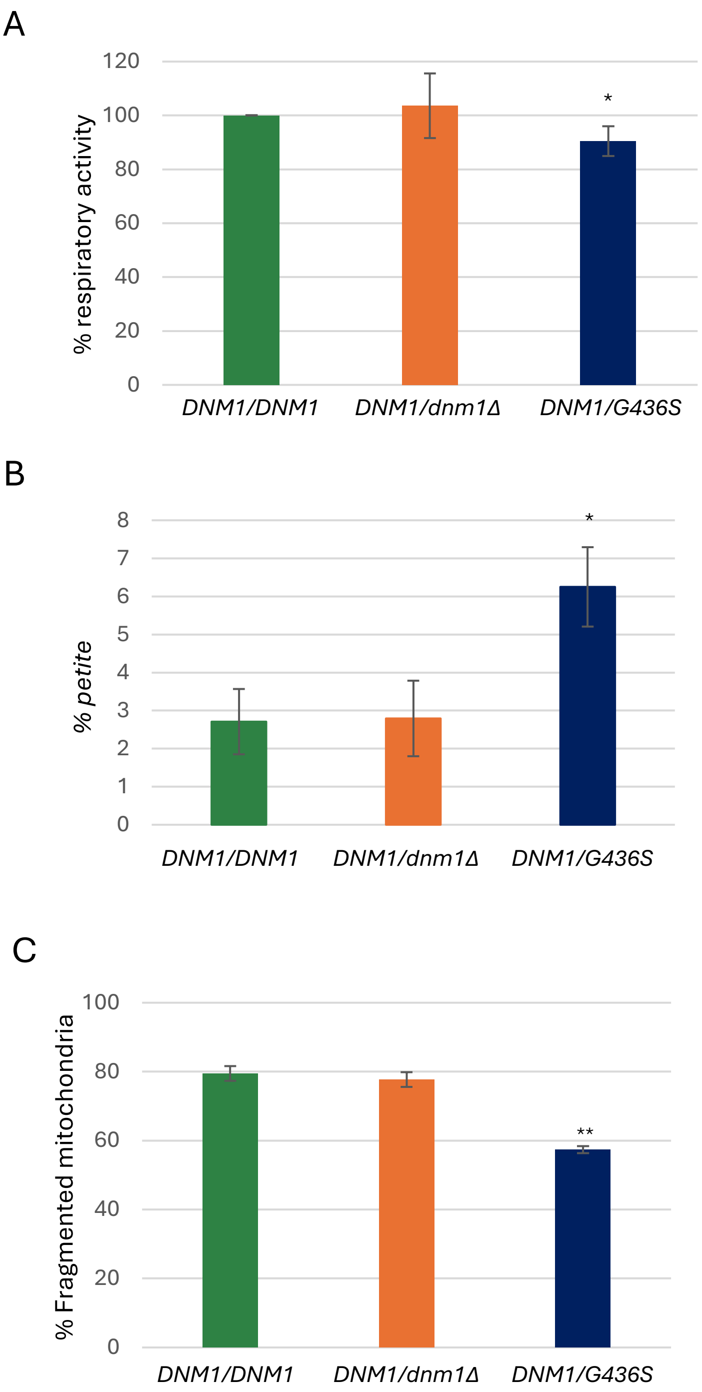

Supplementary Figure S3. **Additional analyses in the diploid yeast *S. cerevisiae***
A. Oxygen consumption rate of the diploid *DNM1/dnm1Δ* strains harboring wild-type *DNM1* (dark green), the empty vector (orange) or the mutant allele *dnm1*^{G436S} (dark blue). Cells were grown at 37 °C in SC medium without uracil supplemented with 0.6% glucose till exhaustion. Values are represented as the mean of at least five values ±SD. **B**. *Petite* frequency (±SD) of the same strains as in (A) at 37°C. **C**. Mitochondrial morphology of the same strains as in (A) after treatment with sodium azide. For each strain, the percentage of cells (± SD) showing fragmented mitochondria is reported.
Statistical analysis was performed using a one-way ANOVA followed by Bonferroni's post-hoc test: * $p < 0.05$; ** $p < 0.01$.

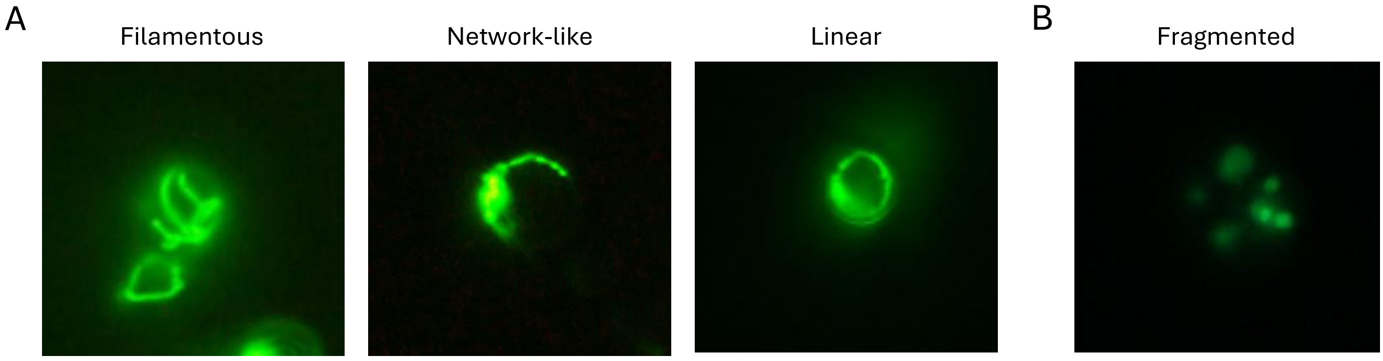

Supplementary Figure S4. **Representative visualization of mitochondrial network/fragmentation.**
A. Yeast strains transformed with mtGFP and classification of the mitochondrial network in three morphotypes: filamentous (long and branched mitochondria); network-like (fused filamentous mitochondria forming network-like structures) and linear (long, unbranched mitochondria). **B.** Representative image of the fragmented mitochondrial network induced by sodium azide treatment.
